# Association between personality factors and health-related quality of life in type 1 diabetes patients

**DOI:** 10.20945/2359-3997000000524

**Published:** 2022-10-11

**Authors:** Maria Luiza Nogueira de Barreiros Gavazza, Eduardo Martins, Ana Claudia Rebouças Ramalho

**Affiliations:** 1 Universidade Federal da Bahia Faculdade de Medicina da Bahia Salvador BA Brasil Universidade Federal da Bahia, Faculdade de Medicina da Bahia, Salvador, BA, Brasil; 2 Universidade Federal da Bahia Unidade de Vigilância Sanitária do Hospital Universitário Professor Edgar Santos Salvador BA Brasil Universidade Federal da Bahia, Unidade de Vigilância Sanitária do Hospital Universitário Professor Edgar Santos, Salvador, BA, Brasil; 3 Universidade Federal da Bahia Faculdade de Medicina da Bahia Departamento de Medicina Interna e Apoio ao Diagnóstico Salvador BA Brasil Universidade Federal da Bahia, Faculdade de Medicina da Bahia, Departamento de Medicina Interna e Apoio ao Diagnóstico, Salvador, BA, Brasil

**Keywords:** Type 1 diabetes, personality, quality of life

## Abstract

**Objectives:**

The objective of the present study was to evaluate a possible association between personality factors (PF) and the health-related quality of life (HRQoL) of type 1 diabetes (T1D) patients. This allows for the investigation of obstacles related to treatment type and the presence of complications in HRQoL.

**Materials and methods:**

This cross-sectional study enrolled 78 patients aged 13-67 years from two diabetes clinics. PF was evaluated using the validated questionnaire Inventory of the Five Great Personality Factors. HRQoL was determined using the Brazilian Problem Areas in Diabetes Scale (B-PAID) questionnaire. The chi-square test, Fisher's exact test, and Welch's modified two-sample t-test were used to establish relationships.

**Results:**

In this sample of 46 women and 32 men with T1D and mean A1C of 8%-9%, we observed great suffering in 58.97% and that HRQoL was worse in women. “Openness” was the most prevalent PF and “extroversion” the least prevalent. “Neuroticism” facilitated a tendency to tolerate suffering.

**Conclusion:**

T1D patients’ personalities influence their treatment. The PF “neuroticism” is potentially related to better HRQoL. Brazilian T1D patients indicated great suffering in their HRQoL, which may be characteristic across the country. Women experienced worse HRQoL, which is in line with world literature. However, the limited sample size in this study warrant further research to test the hypotheses.

## INTRODUCTION

Of the different types of diabetes that have been identified and classified, the most prevalent are type 1 diabetes (T1D) and type 2 diabetes (T2D; 1). Effective treatment for T1D was concretely established in the cohort of the Diabetes Control and Complications Trial (DCCT; 1–3), which evidenced the necessity of intensive insulin therapy for the prevention of chronic complications of the disease. This trial established the need for an adequate basal-bolus insulin regimen, capillary blood glucose monitoring, carbohydrate counting, and diabetes education for T1D (4), now recommended by national and international guidelines (5,6). Also necessary are daily care measures such as carbohydrate counting, multiple daily insulin doses (MDI) or use of an insulin pump, capillary blood glucose monitoring, or use of blood glucose sensors for management of hyper- and hypoglycemia (5,6).

Although studies on the intensive treatment of patients with T1D have shown the importance of optimizing successful disease control (3,4), adherence to this complex therapeutic routine is affected by factors both internal and external to the patients. The impact of the chronic nature of T1D on patients’ lives has been investigated and reported (7). Treatment is influenced by diabetes education, age, sex, socioeconomic status, and family support (8–12). In addition to these elements, patients’ personality factors seem to affect the process of adapting to treatment regimens and, consequently, therapeutic adherence, as found in a study conducted in the United Kingdom (13).

The present study examines patients’ quality of life through the framework of the World Health Organization (14), based on the individual's perception of their position in life; the cultural context and value system in which they live; and their goals, expectations, standards, and concerns. This perspective includes interference from the environment in which the subject's environment and its reflection in their self-assessment. Thus, it places the individual as an active agent in the process of obtaining quality of life.

The concept of quality of life is subdivided when it is assessed. According to Guayatt and cols. (15), an example of this subdivision is health-related quality of life (HRQoL), “a measure of the patient's individual subjective opinion considering their health, in physical, psychological and social dimensions.” This article uses the concept of specific HRQoL for T1D described by W. H. Polonsky (15,16).

The association between PF and the repercussions on HRQoL of patients with T1D still requires further study, justifying the present research.

Therefore, the objective of this study is to investigate associations between PF and HRQoL by evaluating the predominant PF, the treatment performed (MDI and insulin pump), and the presence of T1D complications.

Considering psychology as a central element rather than merely supporting treatment, this study aims to contribute to revising the therapeutic process for patients with T1D.

## MATERIALS AND METHODS

This is an exploratory, quantitative, yearlong, cross-sectional study investigating the general frequency of different personalities among patients with T1D from two different settings: the public research institution Public Health Assistance (PHA) and a private clinic (PC), both located in Salvador, Bahia, Brazil. The two settings are focused on T1D and include assessments to correlate personality factor with quality of life related to T1D. Due to the exploratory nature of the study, sample *n* was not calculated; however, we deemed a minimum *n* of 10 participants per PF subgroup to be satisfactory, resulting in a minimum *n* of 50 – though recognized as a difficult variable to analyze. In this study, we were unable to obtain the data of three selected patients.

The period for data collection was set at one year. We proposed that each patient be given 15 minutes to complete the forms in a private room in the presence of one of the principal researchers (Dr. Ana Claudia Ramalho and Maria Luiza Gavazza).

The approach to the questionnaires was to give each participant two copies of the Free and Informed Consent Form, previously approved by the Professor Edgard Santos University Hospital Complex Ethics Committee (CEP HUPES) as number 3.319.055 at the institution. In the case of children under 18 years, two copies of the Informed Consent Form were also provided.

Inclusion criteria were:

- Children, preadolescents, adolescents, and adults diagnosed with T1D, ages 13 to 67, who were patients of either PHA or PC, and
- Were treated using a multiple daily injections (MDI) scheme or used an insulin pump, and
- Performed carbohydrate counting, and
- Monitored capillary blood glucose more than three times a day or used a blood glucose sensor.

The exclusion criteria were:

- Patients under 13 years old and over 67 years old; or
- Patients diagnosed with decompensated mental disorder. In our data, one patient had a psychiatric medical record establishing generalized anxiety disorders; or
- Patients with thyroid diseases and hypocortisolism or hypercortisolism, currently decompensated; or
- Patients who refused to participate in the research.

The data collected initially were: full name, medical record from the clinic, sex, age, last A1C result, year of T1D diagnosis, presence of related complications (if confirmed, which?), and use of an insulin pump. These data were obtained from the patients’ medical records.

After identifying the participants, we conducted two questionnaires.

### First questionnaire

The Brazilian Portuguese version of the Inventory of the Five Great Personality Factors (IGFP-5), created by John, Danawe and Kentue in 1991 and adapted by Andrade (17), recommended approaching people over age 13 and under age 67, limitations that were applied to the present study. The IGFP-5 contains 44 statements about self-perception and how the individual acts in certain situations, which must be answered using a five-point Likert scale ranging from 1 (*strongly disagree*) to 5 (*strongly agree*). This scale assesses personality dimensions without taking into account individual facets, and is able to separate individuals according to the five PFs of openness, conscientiousness, extroversion, kindness, and neuroticism. The definitions adopted adhere to the following classifications:

1. Openness: Individuals with a high score in this dimension are generally frank, imaginative, witty, original, and artistic.
2. Conscientiousness: Conscientious individuals are generally cautious, trustworthy, organized, and responsible.
3. Extroversion: Extroverted individuals tend to be active, enthusiastic, dominant, sociable, and eloquent or talkative.
4. Kindness: Individuals with high scores in this trait are pleasant, kind, cooperative, and affectionate.
5. Neuroticism: Neurotic individuals are generally nervous, highly sensitive, tense, and concerned (12).

**Figure 1:**
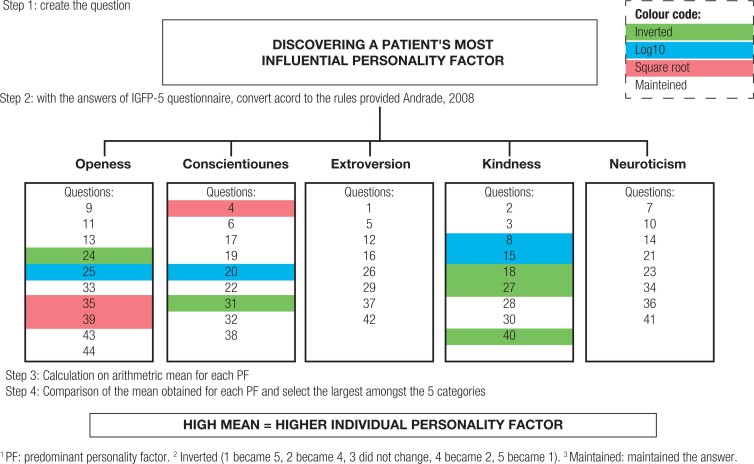
Discernment of FP^1^ in T1D patient, by Andrade, 2008.

We analyzed the response scores for the IGFP-5 questionnaire according to the parameters provided by Andrade (17). Each of the 44 five-point Likert scale responses were analyzed individually by keeping the rating (1–5), replacing it with its square root, replacing it with its inverse (1 being considered the inverse of 5, 2 the inverse of 4, 3 maintained, 4 the inverse of 2, and 5 the inverse of 1), or replacing it with statistical log10 – in such an order that the average of each answer's total within each possible PF was staggered, with the highest result considered the most influential PF in the individual, as illustrated in the flow of acquisition in Figure 1.

As shown in Figure 1, each patient's data relating to the “openness” PF was calculated from the average of their answers to questions 9, 11, 13, 33, 43, and 44 (maintained); 24 (inverted); 25 (log10); and 35 and 39 (square root). The PF “conscientiousness” was the average of the answers to items 4 (square root); 20 (log10); 31 (inverted); and 6, 17, 19, 22, 32, and 38 (maintained). We measured the PF “extroversion” by averaging the participant's answers to questions 1, 5, 12, 16, 26, 29, 37, and 42 (maintained). The PF “kindness” was calculated from the average of the answers to 8 and 15 (log10); 18, 27 and 40 (inverted); and 2, 3, 28 and 30 (maintained). The PF “neuroticism” was determined by the average of the answers to questions 7, 10, 14, 21, 23, 34, 36 and 41 (maintained).

### Second questionnaire

We assessed participants’ quality of life related to diabetes by using the Brazilian version of the Problem Areas in Diabetes Scale questionnaire (B-PAID; 18,19). This quantitatively evaluates the relation of diabetes treatment to its impact on the patient's personal life. The questionnaire involves 20 questions about emotional states commonly reported by patients with T1D and T2D, serving as a predictor of the patient's disease outcome and a tool for clinical treatment analysis. The instrument uses a scale from 0-100; the higher the score, the greater the level of associated emotional distress.

A five-point Likert scale was used (0 = *not a problem*, 1 = *minor problem*, 2 = *moderate problem*, 3 = *somewhat serious problem*, and 4 = *serious problem*). We obtained the final score in the 0-100 range by adding the 0-4 scale answer value for each of the 20 questions and subsequently multiplying these values by 1.25. The questionnaire classifies participants with a total score of ≥ 40 as patients with “great suffering” and those with a score of < 40 as patients with “tolerable suffering” related to diabetes (18,19).

We entered the data into Excel and performed a statistical analysis using the SPSS 18.0 statistical package. Frequency distributions, dispersion summaries (standard deviations), and center measures (mean and median) were calculated for continuous variables. For the nominal and ordinal variables, the distribution of proportions was calculated.

We also used a Likert scale of five aspects (used as a continuous scale) to assess quality of life for T1D patients. For the general group studied, as well as for each personality factor, we calculated the mean and standard deviation. The chi-square test or Fisher's exact test was applied when the expected cell value was less than 5 (for 2 × 2 contingency tables). We also used Welch's modified two-sample t-test, performing a one-sample, two-sample, or Welch's modified two-sample t-test based on user-supplied summary information. The output is identical to that produced with a t-test (20).

For the one-sample t-test, the null hypothesis is that the mean of the population from which *x* is drawn is μ. For the standard and Welch's modified two-sample t-tests, the null hypothesis is that the population mean for *x* less that for *y* is μ. The alternative hypothesis in each case indicates the direction of divergence of the population mean for *x* (or difference of means for *x* and *y*) from μ (20–22).

## RESULTS

### Characterization of the population

The study's population is characterized in Table 1, divided by sex. In Table 2, the population is divided by services.

**Table 1:** General characteristics of the sample in patients with T1D, according to sex

		Men (n = 32)	Women (n = 46)	Prevalence ratio	P-value
Age (years), mean ± SD^1^		24.81 ± 13.47	30.5 ± 13.11	–	0.068***
Time with T1D^2^ (years), mean ± SD		12.0 ± 12.9	13.5 ± 10.9	–	0.593***
A1C^3^ (%), mean ± SD		8.8 ± 2.46	8.38 ± 3.12	–	0.509***
Complications of T1D					
	Patient with one or more complications, *n* (%)	13 (40.63)	19 (41.31)	0.984	0.952*
	Patient without complications, *n* (%)	19 (59.37)	27 (58.69)		
B-PAID^4^, mean ± SD		35.94 ± 22.15	42.09 ± 22.82	–	0.238***
B-PAID, *n* (%)					
	Great suffering, *n* (%)	22 (68.75)	24 (52.17)	1.530	0.143*
	Tolerable suffering, *n* (%)	10 (31.25)	22 (47.83)		
Treatment					
	Insulin pump	8 (25.00)	13 (28.26)	0.905	0.749*
	MDI^5^	24 (75.00)	33 (71.73)		
Using a sensor?					
	Yes	6 (18.8)	13 (28.3)	0.717	0.336*
	No	26 (81.3)	33 (71.7)		
Predominant personality factor, *n* (%)					
	Openness	7 (21.88)	13 (28.26)	–	0.862**
	Conscientiousness	8 (25.00)	9 (19.57)		
	Extraversion	4 (12.50)	7 (15.22)		
	Kindness	8 (25.00)	8 (17.39)		
	Neuroticism	5 (15.63)	9 (19.57)		

1 SD: standard deviation.

2 T1D: type 1 diabetes mellitus.

3 A1C: glycated hemoglobin, *n* = 70.

4 B-PAID: Brazilian Problem Areas in Diabetes questionnaire.

5 Multiple daily injections.

* Chi-squared test.

** Fisher's exact test.

*** Welch's modified two-sample t-test.

**Table 2:** General characteristics of the sample of patients with T1D, according to health service

		PHA^1^ (n = 52)	PC^2^ (n = 26)	Prevalence ratio	P-value
Age (years), mean ± SD^3^		28.48 ± 13.19	27.54 ± 14.26	–	0.780***
T1D^4^ (years), mean ± SD		12.1 ± 11.7	14.5 ± 11.9	–	0.403***
A1C^5^ (%), mean ± SD		8.8 ± 2.54	8.0 ± 1.41	–	0.078***
Complications of T1D					
	Patient with one or more complications, n (%)	25 (48.07)	7 (26.92)	1.331	0.073*
	Patient without complications, n (%)	27 (51.93)	19 (73.08)		
B-PAID^6^, mean ± SD		40.88 ± 23.79	36.94 ± 20.20	–	0.448***
B-PAID					
	Great suffering, n (%)	30 (57.69)	16 (61.54)	0.949	0.746*
	Tolerable suffering, n (%)	22 (42.30)	10 (38.46)		
Treatment					
	Insulin pump	5 (9.61)	16 (61.53)	0.284	< 0.001*
	MDI^7^	47 (90.3)	9 (34.61)		
Using a sensor?					
	Yes	1 (1.9)	18 (69.2)	0.061	< 0.001*
	No	51 (98.1)	8 (30.8)		
Predominant personality factor					
	Openness, *n* (%)	16 (30.77)	4 (15.38)	–	0.309**
	Conscientiousness, *n* (%)	8 (15.38)	9 (34.62)		
	Extraversion, *n* (%)	8 (15.38)	3 (11.54)		
	Kindness, *n* (%)	10 (19.23)	6 (23.08)		
	Neuroticism, *n* (%)	10 (19.23)	4 (15.38)		

1 PHA: Magalhães Neto Ambulatory.

2 PC: private clinic.

3 SD: standard deviation.

4 T1D: type 1 diabetes mellitus.

5 A1C: glycated hemoglobin,

*n* = 70.

6 B-PAID: Brazilian Problem Areas in Diabetes questionnaire.

7 Multiple daily injections.

* Chi-squared test.

** Fisher's exact test.

*** Welch's modified two-sample t-test.

There were 78 patients aged 13-67 years with T1D from two clinics in Salvador: PHA (52) and PC (26), 46 women (59%) and 32 men (41%). The women in the sample were older, with an average age of 30.5 ± 13.1, as seen in Table 1.

We divided the participants by T1D complications, classifying them as “patients with one or more complications” and “patients without complications”. Between sexes, the number of patients with complications was similar: 41.3 and 40.63, respectively, for women and men (Table 1). Of PHA patients, 48.1% had at least one complication of T1D, whereas at PC, 73.1% had no complications (Table 2).

Regarding treatment for T1D, we obtained data on glycemic control from 70 of the 78 patients. Women were associated with lower A1C results (8.38% ± 3.12) than were men (8.8% ± 2.46; Table 1). Differences emerged regarding the predominance of MDI in PHA patients (90.3%), as opposed to the use of an insulin pump by PC patients (61.5%). The mean A1C between treatment centers was 8.8% ± 2.54 v. 8.0% ± 1.41, respectively, at the PHA and PC (Table 2). PC patients tended to have longer disease duration, lower prevalence of diabetes complications, and lower A1C. When we calculated the association of prevalence ratio between sexes, there was no evidence of statistical relevance.

### Quality of life

The B-PAID score classifies patients with ≥ 40 as having “great suffering” and < 40 as patients with “tolerable suffering” related to T1D (13,14).

Among sexes, the B-PAID score was found to be higher in women, suggesting worse HRQoL connected to T1D in women. Though the number of women classified as having T1D-related “great suffering” was similar to the number of women with “tolerable suffering” (24 and 22 patients, respectively), among men there was a predominance of “great suffering” (22 and 10 patients, respectively, classified to have “great” and “tolerable suffering”; Table 1).

The information obtained from the sample showed that patients at both clinics with “great suffering” predominantly related it to T1D (57.7% and 61.5%, respectively, at PHA and PC). The general analysis of the sample showed that patients with “great suffering” represented 59% of the studied population (Table 2). We also calculated the prevalence ratio between services, without statistical relevance associated.

### Personality

The data referring to the personality of the patients are shown in Table 3; details can be obtained from Figure 1. We calculated the representations of each factor in this study's sample, finding that openness was the most prevalent (20 individuals) and extroversion the least prevalent (11 individuals).

**Table 3:** Correlation between FP^1^ and HRQoL^2^ (by B-PAID^3^) in patients with T1D^4^

	Personality factor					
	Opening	Conscientiousness	Extroversion	Kindness	Neuroticism	P value
Score B-PAID, mean ± SD^5^	38.9 ± 22.5	36.8 ± 20.6	34.9 ± 23.0	39.8 ± 25.3	47.2 ± 23.0	0.63*
Great suffering, *n* (%)	12 (60)	10 (58.8)	7 (63.6)	10 (62.5)	7 (50)	0.66*
Tolerable suffering, *n* (%)	8 (20)	7 (41.2)	4 (36.4)	6 (37.5)	(50)	

1 PF: predominant personality factor, obtained from Figure 1.

2 HRQoL: health-related quality of life.

3 B-PAID: questionnaire of Brazilian Problem Areas in Diabetes.

4 T1D: type 1 diabetes mellitus.

5 SD: standard deviation.

* Fisher's exact test.

We found no statistically significant difference between PF and service, sex, or HRQoL.

No statistical significance was identified for the neuroticism PF for most patients with tolerable suffering. There was a trend with statistical significance associating most patients with the PFs openness and kindness with T1D-related “great suffering”.

## DISCUSSION

Our results showed that almost 60% of the sample was classified as having “great suffering” related to T1D, mainly associated with the predominance of the PFs openness and kindness.

Considering the objective of this study – to search for an association between PFs and HRQoL in patients with T1D – the results obtained are in agreement with a study from the United Kingdom by Rassart and cols. (13). The authors used the five-factor model of personality and the PAID questionnaire to evaluate the influence of personality on the adjustment to treatment for individuals with T1D, concluding that personality does influence treatment. In our data, we found a tendency associating T1D-related “tolerable suffering” and patients with neuroticism; we also observed a tendency for T1D patients with extroversion and kindness to have worse HRQoL. This correlation was not previously described in the literature.

Contrary to our data, Huang and cols. (23) performed a systematic review of personality and quality of life associated with chronic diseases, finding that the neuroticism factor was related to worse quality of life, and that extroversion and kindness PFs pointed to a better quality of life. The review contains only one article about patients with T1D and T2D and assessed non-specific quality of life for those with the disease. This study's findings are associated specifically with the characteristics of T1D, a particular chronic disease that requires patients to strategically organize their lives to obtain positive treatment results. Thus, patients with the PF neuroticism could be more involved in the above-mentioned aspect of treatment, which would justify their better HRQoL related to T1D. Conversely, the correlation between the PFs extroversion and kindness with worse HRQoL of the patients with T1D could be related to a less focused and less demanding patient profile regarding the treatment itself.

A Brazilian study by Calliari and cols. (24) evaluating the use of the Free Style Libre^®^ sensor for blood glucose monitoring found that the daily number of blood glucose screenings is related to A1C. The national screening average was 14 times a day. Patients who screened an average of 43.1 times a day obtained an A1C level of 6.71%, whereas those who screened an average of 3.6 times obtained an A1C level of 7.56% (*p* < 0.01). Such a finding suggests that a personality more attentive to details and more engaged in the treatment of T1D could explain the screening frequency above the national average and better levels of A1C. These characteristics of patients are consistent with the PF neuroticism, which could explain the results observed in this study showing better HRQoL for T1D patients associated with neuroticism.

An Australian study (25) correlated chronic diseases and better quality of life in the country. In another study, also in Australia (26), a clinic using the PAID questionnaire to assess HRQoL of T1D, found that 29.3% of patients with T1D were in great distress, whereas this study's results found almost twice this amount (58.97%). This may suggest the interference of the health model adopted in each country, as well as of the characteristics of the national population. In agreement with this study's results, Martins and cols. (27) used the Quality of Life Instrument for Young People with Diabetes adapted to Brazilian patients (IQVJD) to evaluate the HRQoL of T1D patients in Brazil and found that more than 50% had low HRQoL. Thus, the substantial impact of T1D on the lives of Brazilians is reinforced and seems to be characteristic of the country.

The current study's results also found that women with T1D tend to have worse HRQoL, which is in line with a study conducted in the United Kingdom using the same PAID questionnaire (28). This could potentially be explained by the unfavorable position of women in society resulting from cultural and family contexts, and was discussed in a Latin American study (29).

The possible limitations of our research were related to the characterization of the population: participants’ body mass indices were not obtained but may have an impact on glycemic control, and we did not use variable glycemic control to correlate with PFs due to the lack of robust data on glycemic control in the sample, especially for PHA patients. In addition, a study performed in one city with a limited number of patients may not reflect the rest of the country or T1D patients in general; therefore, additional studies on the same topic are necessary to confirm our hypothesis. In sum, our study was limited by the number of participants *n*; limited area in a city of Brazil; and the difficulty of obtaining an A1C average or *time in range*, mainly in PHA. In addition, we were unable to correlate glycemic control with personality factors, as the available A1C data are insufficient (30) to faithfully represent glycemic control in T1D.

In conclusion, we conclude that women with T1D have worse HRQoL and this is in agreement with the literature on the topic worldwide. We showed that personality seems to influence the HRQoL of patients with T1D, and that PF neuroticism is not associated with worse HRQoL in T1D.

Statistics also show that the HRQoL of patients with T1D in Brazil indicates “great suffering” and that this result may be characteristic of the entire country. Therefore, more studies are needed to reinforce our results.
